# The ChEMBL database in 2017

**DOI:** 10.1093/nar/gkw1074

**Published:** 2016-11-28

**Authors:** Anna Gaulton, Anne Hersey, Michał Nowotka, A. Patrícia Bento, Jon Chambers, David Mendez, Prudence Mutowo, Francis Atkinson, Louisa J. Bellis, Elena Cibrián-Uhalte, Mark Davies, Nathan Dedman, Anneli Karlsson, María Paula Magariños, John P. Overington, George Papadatos, Ines Smit, Andrew R. Leach

**Affiliations:** 1 European Molecular Biology Laboratory, European Bioinformatics Institute, Wellcome Genome Campus, Hinxton, Cambridgeshire CB10 1SD, UK; 2 Open Targets, Wellcome Genome Campus, Hinxton, Cambridgeshire CB10 1SD, UK

## Abstract

ChEMBL is an open large-scale bioactivity database (https://www.ebi.ac.uk/chembl), previously described in the 2012 and 2014 Nucleic Acids Research Database Issues. Since then, alongside the continued extraction of data from the medicinal chemistry literature, new sources of bioactivity data have also been added to the database. These include: deposited data sets from neglected disease screening; crop protection data; drug metabolism and disposition data and bioactivity data from patents. A number of improvements and new features have also been incorporated. These include the annotation of assays and targets using ontologies, the inclusion of targets and indications for clinical candidates, addition of metabolic pathways for drugs and calculation of structural alerts. The ChEMBL data can be accessed via a web-interface, RDF distribution, data downloads and RESTful web-services.

## INTRODUCTION

Since its inception a major component of ChEMBL's content has been bioactivity data regularly extracted from the medicinal chemistry literature (1,2). Among many other applications such data enables researchers to identify tool compounds for potential therapeutic targets, to probe the available SAR data for a target, investigate phenotypic data associated with similar compounds and to identify potential off-target effects of specific chemotypes. In order to provide a more complete perspective, based in part on user feedback, the scope and data types within ChEMBL have gradually expanded, with some major new areas included in recent releases: compounds in clinical development, data from patents, direct depositions for neglected diseases and agrochemical data.

Drug discovery remains a costly process with a high failure rate (3–6). To provide a more complete picture across the drug discovery and development process, and to help researchers better understand what makes a successful medicine, we have extended the ChEMBL data model to include, for the first time, data typically generated in the pre-clinical and clinical phases of drug discovery, specifically drug metabolism and disposition data. Another common approach to understanding pharmaceutical attrition is to learn from successful drugs and failed drug candidates (7–9). We have therefore extended our set of drug-target annotations to include those for clinical candidates and have also mapped these chemical entities to their therapeutic indications.

At the end of 2013, EMBL-EBI took over the operation, development and support of the SureChem patent system (now called SureChEMBL (10)) from Digital Science Ltd. Access to this resource has highlighted the potential value to scientists of bioactivity data not yet published in the scientific literature. However, the current SureChEMBL system only extracts compound structures from the patents and not associated bioactivity data. As a first step to address this opportunity we have worked with BindingDB to incorporate the BindingDB patent data into ChEMBL (11).

Neglected disease research continues to be a field of drug discovery conducted largely (though not exclusively) by not-for-profit organisations that aim to expedite research by encouraging sharing of experimental data with the community (12–15). Depositions of this type of data into ChEMBL have continued to increase since the original malaria depositions in 2010.

Whilst the pharmaceutical and drug discovery community continues to be the major user and consumer of ChEMBL data, other life sciences communities also work with similar types of data. The agrochemical industry is one such community where specific efforts have been made to widen the coverage of data relevant to the discovery and development of herbicides, pesticides and fungicides (16).

In the next sections, we describe the new data types now integrated into the ChEMBL database, the annotations we have undertaken to enable structured organisation and access to the data and how the new data and annotations can be viewed via the web interface.

## DATA CONTENT

### Current data content

ChEMBL's content continues to grow; release 22 of the database contains information extracted from more than 65 000 publications, together with 50 deposited data sets, and data drawn from other databases (Table 1). In total, there are >1.6 million distinct compound structures represented in the database, with 14 million activity values from >1.2 million assays. These assays are mapped to ∼11 000 targets, including 9052 proteins (of which 4255 are human).

**Table 1.** Data sources included in the ChEMBL release 22

Short name	Source	No. compounds	No. assays	No. activities
LITERATURE	Scientific Literature	967 242	963 186	5 635 084
PUBCHEM_BIOASSAY	PubChem BioAssays	489 575	2937	7 559 601
GATES_LIBRARY	Gates Library compound collection	68 490	2	69 444
BINDINGDB	BindingDB Database	68 149	1317	99 061
GSK_TCMDC	GSK Malaria Screening	13 467	6	81 198
ST_JUDE_LEISH	St Jude Leishmania Screening	13 422	6	42 105
USP/USAN	USP Dictionary of USAN and International Drug Names	11 356	0	0
DNDI	Drugs for Neglected Diseases Initiative (DNDi)	7053	233	14 452
ASTRAZENECA	AstraZeneca Deposited Data	5799	15	11 687
NOVARTIS	Novartis Malaria Screening	5614	6	27 888
ORANGE_BOOK	Orange Book	2016	0	0
SUPPLEMENTARY	Deposited Supplementary Bioactivity Data	1786	13	4817
CANDIDATES	Clinical Candidates	1633	0	0
ST_JUDE	St Jude Malaria Screening	1524	16	5456
TP_TRANSPORTER	TP-search Transporter Database	1434	3592	6765
DRUGMATRIX	DrugMatrix	930	113 678	350 929
METABOLISM	Curated Drug Metabolism Pathways	828	0	0
GSK_TB	GSK Tuberculosis Screening	826	15	1814
WHO_TDR	WHO-TDR Malaria Screening	740	16	5853
GSK_TCAKS	GSK Kinetoplastid Screening	592	13	7235
MMV_MBOX	MMV Malaria Box	400	138	45 158
MMV_PBOX	MMV Pathogen Box	400	0	0
ATLAS	Gene Expression Atlas Compounds	398	0	0
DRUGS	Manually Added Drugs	378	0	0
GSK_PKIS	GSK Published Kinase Inhibitor Set	366	456	169 451
OSM	Open Source Malaria Screening	211	22	344
WITHDRAWN	Withdrawn Drugs	192	0	0
TG_GATES	Open TG-GATEs	160	158 199	158 199
SANGER	Sanger Institute Genomics of Drug Sensitivity in Cancer	137	714	73 169
FDA_APPROVAL	FDA Approval Packages	43	1386	1387
HARVARD	Harvard Malaria Screening	37	4	111

### Deposited data sets

ChEMBL continues to receive data sets from both not-for-profit and commercial organisations that wish to share data with the scientific community. These deposited data sets contain many novel chemical structures and associated bioactivity data. A good example is a library of small molecular weight (∼320 Da average) natural product-like compounds created at the University of Dundee with funding from the Gates Foundation. This library is being screened in a number of neglected disease assays. To date, information about the compound structures and their activity in cytotoxicity assays is available in ChEMBL; further assay data will be deposited as the screening is completed. Another example is the Malaria Box Compound Set (http://www.mmv.org/research-development/open-access-malaria-box), a set of 400 compounds with antimalarial activity that was made available by the Medicines for Malaria Venture (MMV) for research groups to request and screen (15). The latest ChEMBL releases include results from an additional 19 laboratories that have screened and deposited their results on these compounds. The Malaria Box was recently superseded by the Pathogen Box (http://www.pathogenbox.org), which is a new set of compounds with activity against a broader range of pathogens, also available from MMV for screening. These compounds have also been deposited in the ChEMBL database and experimental data will also be added as it becomes available. Four new sets of screening results from the Drugs for Neglected Diseases Initiative (DNDi, http://www.dndi.org) have also been incorporated into recent ChEMBL releases, providing data on compounds tested as potential drugs for neglected diseases such as Leishmaniasis and Chagas disease.

Beyond the area of neglected diseases, the University of Vienna and Roche have deposited supplementary data associated with publications already in ChEMBL (17,18). This is complementary to the similar sets already deposited by GlaxoSmithKline and we encourage similar depositions from other authors. AstraZeneca have taken a different approach to direct data deposition. They identified compounds already in ChEMBL and then provided data on these compounds from a variety of *in vitro* ADME and physicochemical screens including protein binding, microsome and hepatocyte clearance, solubility, p*K*_a_ and lipophilicity. It is important to note that for all such deposited data sets, ChEMBL provides a DOI so that the data can be cited in subsequent publications. For example, the DOI for the AstraZeneca deposited data is 10.6019/CHEMBL3301361 and this resolves to the ChEMBL Document Report Card for the complete data set.

Deposited data sets can be identified through the ChEMBL interface by selecting the relevant entry in the ‘Activity Source Filter’ (located to the right of the keyword search bar) and then performing a keyword search with a wildcard (*) against ‘Documents’ or ‘Assays’. This will return all documents or assays associated with that source or depositor, from which point further information regarding compounds, targets and activity measurements can be navigated.

### Crop protection data

In order to broaden the utility of ChEMBL in crop protection research, a data set of >40 000 compounds and 245 000 activity data points has been extracted from crop protection-related publications and added to ChEMBL (16). This data set significantly increased the content of pesticide, herbicide and insecticide assays in the database. The ChEMBL taxonomy tree browser has been extended to allow easier retrieval of this data (https://www.ebi.ac.uk/chembl/target/browser). In addition, known pesticides already in ChEMBL were assigned a mechanism of action classification, following the Fungicide Resistance Action Committee (FRAC: http://www.frac.info/publications), Herbicide Resistance Action Committee (HRAC: http://hrac.tsstaging.com/tools/classification-lookup) or Insecticide Resistance Action Committee (IRAC: http://www.irac-online.org/documents/moa-brochure/?ext=pdf) systems.

### Patent data exchange

BindingDB is a database of affinity measurements for small molecules binding to protein targets (11). BindingDB curates data from journals complementary to ChEMBL and also incorporates relevant ChEMBL data. More recently, the BindingDB team have abstracted binding affinity data from granted US patents. We have worked with BindingDB to establish a data exchange mechanism for patent data and now include in ChEMBL the patent data extracted by the BindingDB team (ChEMBL source = 37). Identifiers for these patent documents are now included in the DOCS table as PATENT_ID. Since target information is already carefully manually curated in BindingDB, this information is retained in ChEMBL and simply mapped to the equivalent ChEMBL target. The data is taken from 1,015 granted US patents published between 2013 and 2015 and currently comprises 99 061 bioactivities on 68 149 distinct compounds binding to around 600 distinct targets. Of particular interest to drug discovery scientists is the fact that data often appears in patents earlier than in the traditional medicinal chemistry literature. This patent set contains data on 50 targets for which there was previously no data in ChEMBL and which may therefore represent novel targets of therapeutic interest.

## NEW FUNCTIONALITY

### Richer assay and target annotation

Typical entry points to ChEMBL have predominantly been compound-based or target-based searches. However, more than half of the activity data points in ChEMBL come from functional or phenotypic assays that cannot be assigned a molecular target. Since phenotypic screening is once more becoming commonplace in drug discovery (19), making this wealth of data more accessible is a priority. To this end, we have applied a number of ontologies to the ChEMBL assay and activity data, allowing them to be searched and filtered by cell-line, tissue or assay format, for example.

The BioAssay Ontology (BAO) (20,21) was chosen as a means of annotating ChEMBL assays for a number of reasons: this ontology has been developed specifically for small molecule screening data and so provides good coverage of the ChEMBL data; it has also been adopted by a number of other bioassay data providers and members of the drug discovery community, allowing for good data interoperability. ChEMBL standard activity types were manually mapped to corresponding BAO result terms (stored in the ACTIVITIES table as BAO_ENDPOINT). The resulting mappings cover 91% of the activity data points in ChEMBL. The remainder are mainly diverse phenotypic endpoints that are not covered by BAO (e.g. ‘Tissue Severity Score’, ‘Anticonvulsant activity’, ‘Paw swelling’, ‘Relative uterus weight’) or imprecise terms (e.g. ‘Ratio’, ‘Selectivity’, ‘Response’) that require further resolution. Similarly, ChEMBL standard activity units were mapped to Units Ontology (UO) terms (22) (which are also a component of BAO) and stored in the ACTIVITIES table as UO_UNITS. Where available, units were also mapped to terms from the Quantities, Units, Dimensions and Types ontology (http://www.qudt.org). These are stored in the ACTIVITIES table as QUDT_UNITS. The current mapping to Units Ontology covers 87% of ChEMBL activity data points (the remainder largely being complex units e.g. ‘ng.h.ml^-1^’, ‘ml.min^-1^.g^-1^’ that are not covered by UO).

ChEMBL assays have also been annotated with BAO assay format terms, allowing users to distinguish biochemical, cell-based, tissue-based or organism-based assays. An automated, rule-based approach was used to classify the assay format for historical assays, based on information in assay descriptions and target assignments. In order to minimise false assignments, any assays where the format could not be determined unambiguously were left unclassified, pending further curation (11% of assays). The assay format is stored in the ASSAYS table as BAO_FORMAT. Complementary to this, we have also introduced annotation of the cells and tissues used in assays (stored as CELL_ID, ASSAY_CELL_TYPE, TISSUE_ID and ASSAY_TISSUE in the ASSAYS table). A ChEMBL CELL_DICTIONARY and TISSUE_DICTIONARY have been introduced and cell and tissue Report Card pages and search functionality created. It should be noted that the aim here is not to create new cell-line/tissue ontologies – entries in the ChEMBL dictionaries are imported from established vocabularies and ontologies wherever possible (e.g. Cell Line Ontology (23), Experimental Factor Ontology (EFO) (24), Cellosaurus (http://web.expasy.org/cellosaurus/) and LINCS cell dictionary (25) for cells; and Uberon (26), EFO, Brenda Tissue Ontology (27) and CALOHA for tissues (ftp://ftp.nextprot.org/pub/current_release/controlled_vocabularies/caloha.obo)) and mappings to these ontologies are provided.

Methods for browsing and retrieving protein target data in ChEMBL have also been enhanced. Improvements have been made to the existing protein family classification for ion channels and transporters to more closely align the ChEMBL system with other resources (the IUPHAR/BPS Guide To PHARMACOLOGY (28) and TCDB (29)) and new classes have been introduced for epigenetic regulators (following the ChromoHub database (30)). In order to allow browsing of ChEMBL targets by Gene Ontology terms, a new GO Slim has been created (31). This is a subset of GO terms that are enriched in ChEMBL targets and allows the implementation of a simplified Gene Ontology Tree for browsing (on the ‘Browse Targets’ tab).

**Figure 1.**
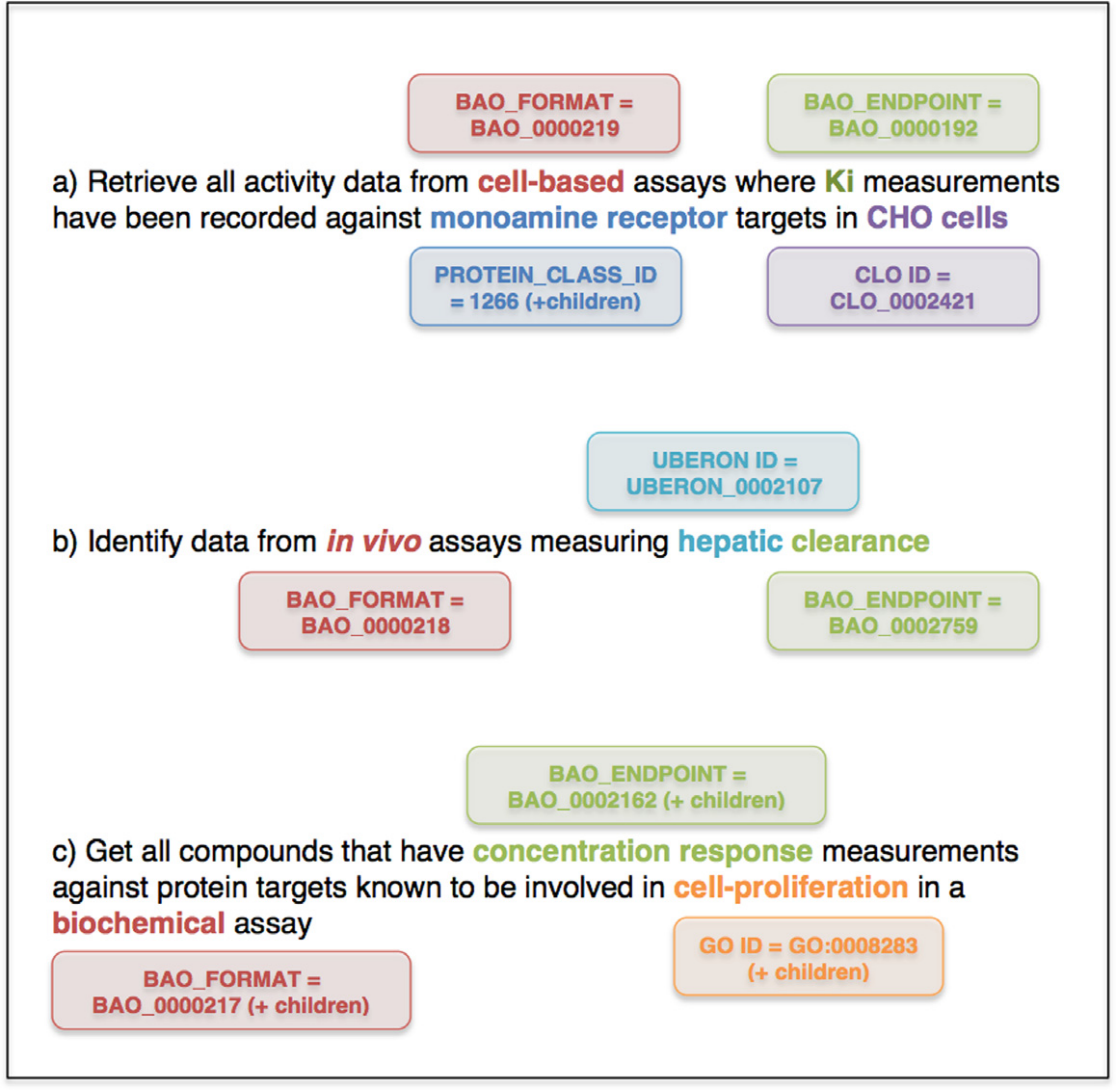
Examples of more complex queries that could be performed (e.g. using web services) by combining BioAssay Ontology, protein family and GO classifications.

These new features, particularly when combined, enable more sophisticated queries of the ChEMBL data to be performed, as illustrated in Figure 1.

### Drug indications and clinical candidates

In previous releases of ChEMBL we annotated properties and efficacy targets for FDA approved drugs. We are now also incorporating drug indication information into ChEMBL to facilitate use-cases such as target validation (the existence of a drug that acts through a particular target and is prescribed for a given indication may be taken as evidence validating the relevance of that target for the indication). Drug indication data is collected from a variety of sources including DailyMed package inserts (https://dailymed.nlm.nih.gov), Anatomical Therapeutic Chemical (ATC) classification (http://www.whocc.no/atc_ddd_index/) (32) and ClinicalTrials.gov (https://clinicaltrials.gov) (33). Since these resources mainly provide free text information, a combination of text-mining (using BeCAS (34)), automated mapping and manual curation/validation is used to identify the indication terms and assign the corresponding disease terms in the Medical Subject Headings (MeSH) vocabulary (https://www.nlm.nih.gov/mesh/) and EFO (24). Two new tables have been added to the database schema to capture this information: DRUG_INDICATION and INDICATION_REFS. A new tab has been added to the ChEMBL interface to display the data—‘Browse Drug Indications’. This view contains a list of all approved drugs and clinical candidates for which indication information is available, and displays the MeSH and EFO terms for the indications and the highest development phase at which each indication has been investigated. The view can be searched and filtered by typing keywords in the ‘Search’ box (e.g. ‘diabetes’ would filter the display to only those drugs that have an indication containing the word diabetes). More specific searches can be performed by entering a MeSH or EFO ID in the search box (e.g. ‘EFO:0001360’ would return drugs indicated for ‘type II diabetes mellitus’). The whole or filtered data set can also be exported using the ‘Downloads’ option at the top right-hand side of the view. A summary of the indication data is also displayed on Compound Report Card pages for drugs.

Furthermore, we are now extending the annotation of efficacy targets and indications to cover drug candidates in clinical development. Mechanism of action and target information for 1,023 clinical candidates in phase I-III development (mainly targeting GPCR, kinase, nuclear hormone receptor and ion channel targets, which are the focus of the NIH-funded Illuminating the Druggable Genome project: https://commonfund.nih.gov/idg/) has already been curated and included in the database (ChEMBL source = 8), and targets for further candidates will be included in future releases. Indications for candidates have also been included using information in ClinicalTrials.gov. For each candidate-indication association, the highest development phase at which that indication has been investigated is also recorded. Currently 1,451 clinical candidates have associated indication information. Clinical candidates are now included in the ‘Browse Drugs’ tab on the ChEMBL interface and their mechanism of action and indication information (where annotated) can be viewed in the ‘Browse Drug Targets’ and ‘Browse Drug Indications’ tabs, respectively.

**Figure 2.**
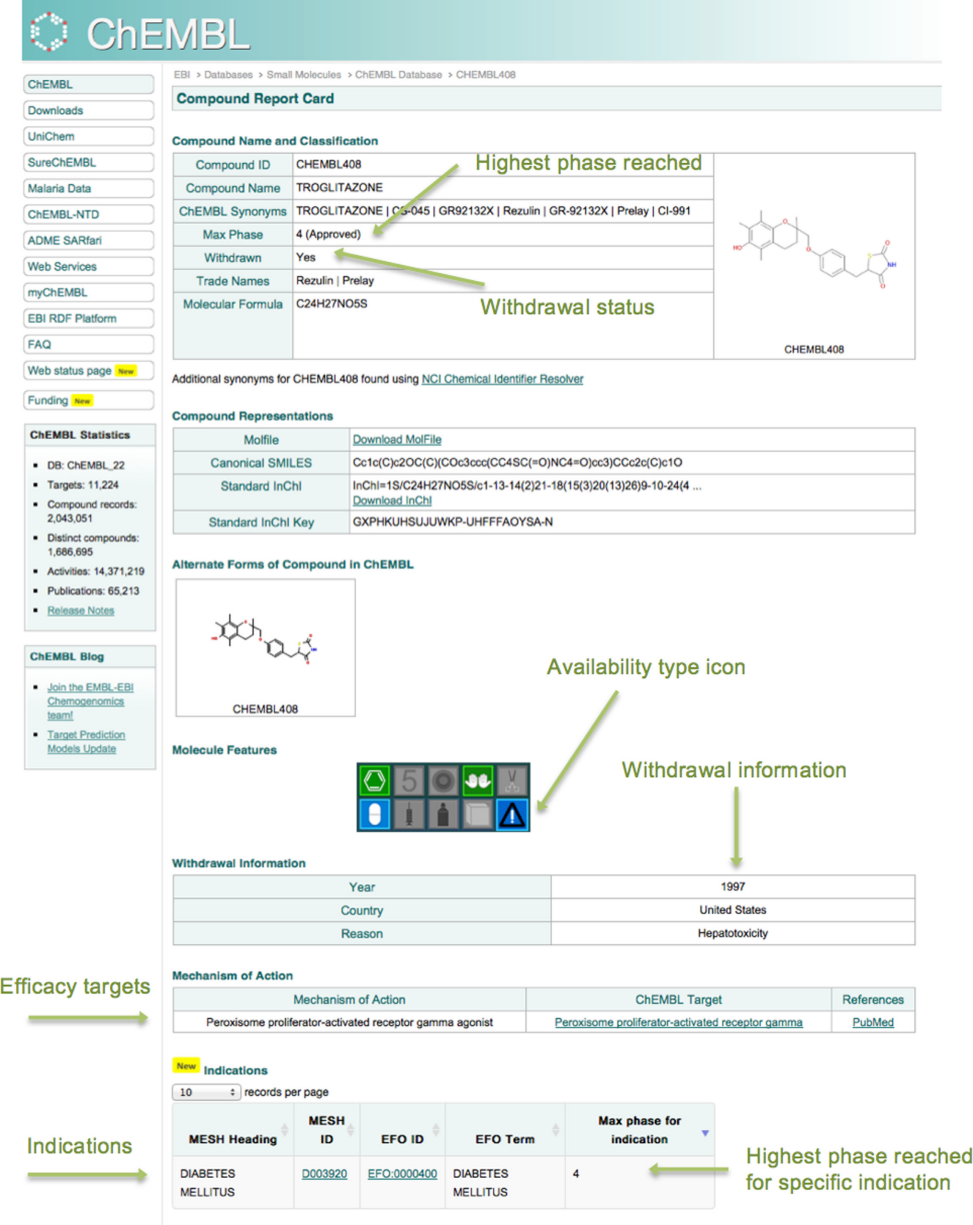
Compound Report Card for Troglitazone showing mechanism of action, indication and withdrawal information (https://www.ebi.ac.uk/chembl/compound/inspect/CHEMBL408).

Finally, we have also added information regarding previously approved drugs that have been withdrawn for toxicity or efficacy reasons. Information regarding withdrawn drugs was collated from several sources: the FDA (http://www.fda.gov) and EMA (http://www.ema.europa.eu/ema/), the WITHDRAWN database (35), the US Electronic Code of Federal Regulations (http://www.ecfr.gov/cgi-bin/retrieveECFR?gp=2&SID=915cc9ab8176f1d1a2a355acf064ffe3&h=L&mc=true&n=sp21.4.216.b&r=SUBPART&ty=HTML#se21.4.216_124), Federal Register (https://www.gpo.gov/fdsys/pkg/FR-2014-07-02/pdf/2014-15371.pdf) and several review articles (36–38). Where available, the year of withdrawal, the applicable countries/areas and the reasons for the withdrawal are captured. This information has been added to the MOLECULE_DICTIONARY database table and is displayed on the ‘Browse Drugs’ tab and also on Compound Report Card pages (see Figure 2). The ‘Molecule Features’ icons have also been updated to include a new availability type icon. This icon now has four options: withdrawn, discontinued, prescription-only, over-the-counter. In cases where the drug has been withdrawn in one country but is still available in others, the icon will retain the prescription-only or over-the-counter status, rather than withdrawn.

### Drug metabolism, toxicity and pharmacokinetic data

*In vivo* data can be extremely valuable but is also invariably complex, with experimental parameters typically being measured at varying doses, time points, routes of administration, in the presence of interacting compounds etc. In order to organise this information in a more structured way and to enable easier access of this information by ChEMBL users an ASSAY_PARAMETERS table has been included in the ChEMBL schema to record this information. This has been done retrospectively for all the ChEMBL assays and resulted in addition of 3.6 million parameter mappings for approximately one third of all the ChEMBL assays.

Additionally, new information has been added to ChEMBL on drug metabolism, pharmacokinetics and toxicology particularly for pre-clinical and clinical drug candidates. The data has been manually extracted from three new data sources and will be extended in future ChEMBL releases. Firstly ∼260 articles covering the years 2011–2013 were extracted from the Journal of Drug Metabolism and Disposition. This resulted in data on ∼2200 compounds tested in nearly 15 000 assays (included in ChEMBL source = 1). The second data set included are the FDA drug approval packages (39). These are summaries of the information submitted to the FDA when the drug was approved for use and are available for FDA drugs approved after 1997. To our knowledge this valuable data is not captured in a structured format elsewhere (ChEMBL source = 28).

**Figure 3.**
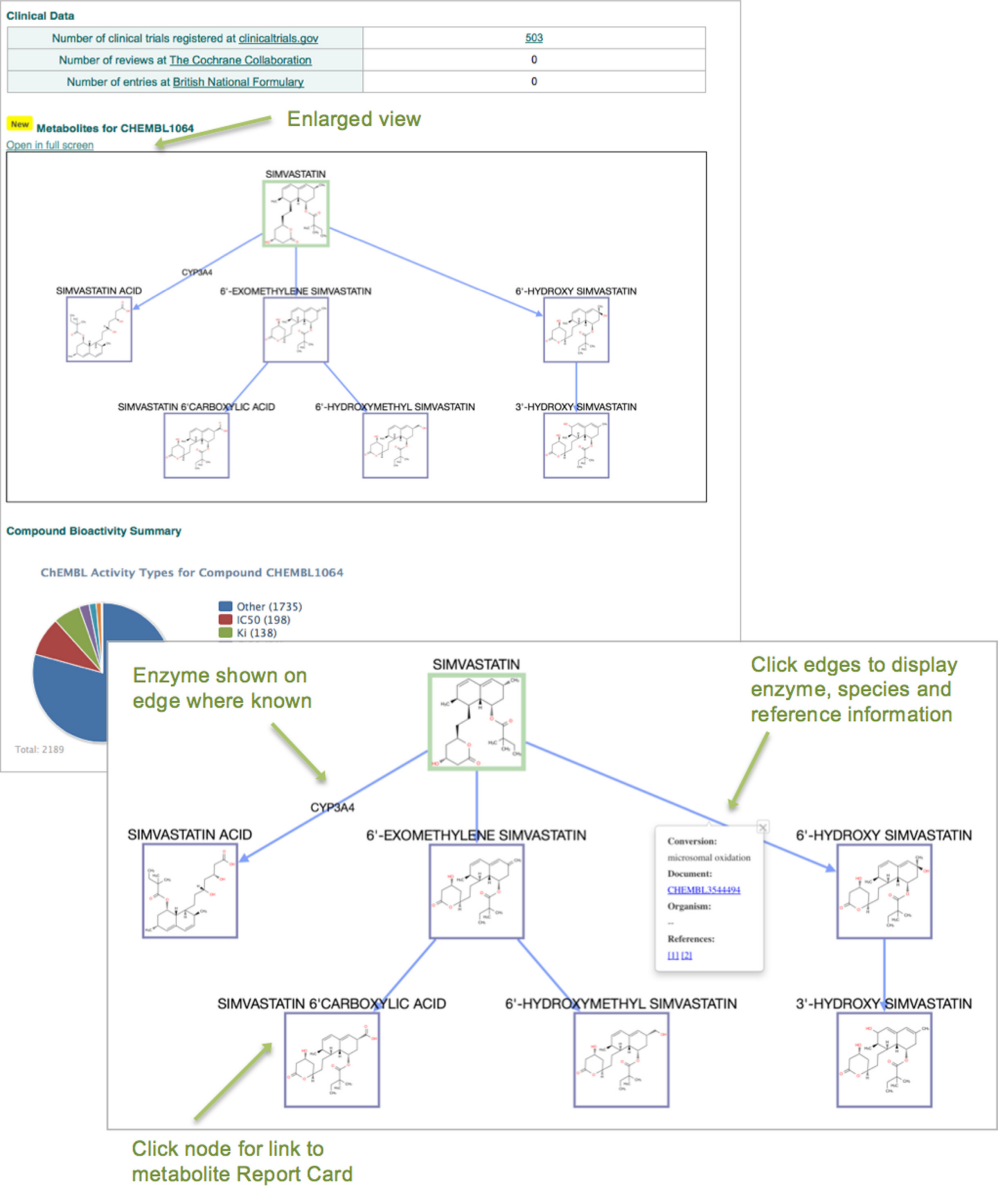
Metabolism scheme for Simvastatin (https://www.ebi.ac.uk/chembl/compound/inspect/CHEMBL1064).

Thirdly, given the need to understand the nature of drug metabolites and whether they themselves are therapeutically active or constitute reactive species that bind DNA or proteins resulting in toxicity (40,41), a project has been initiated to identify the metabolic pathways of approved drugs. This was achieved using a variety of data sources (ChEMBL sources 1, 28 and 31). The metabolic schemes are described in a data model that maintains the relationships between the various molecular entities (e.g. metabolite A may be formed directly from drug D, whereas metabolite B may result from the degradation of metabolite A). Where metabolising enzymes, species and tissues are available in the original publication this information is recorded in ChEMBL. In instances where the metabolite structure is known it is recorded as a chemical structure. If the exact structure is unknown the reaction is still recorded but with an undefined structure. The metabolism data is recorded in two new tables METABOLISM and METABOLISM_REFS. The metabolite pathway is shown on the ChEMBL interface as an interactive image with links to the data on the metabolites, the metabolising enzymes and the document sources for the information. An example for Simvastatin (https://www.ebi.ac.uk/chembl/compound/inspect/CHEMBL1064) is shown in Figure 3.

### Compound structural alerts

In order to aid users in the selection of compounds, for example to create screening sets, we have compiled sets of publicly-available structural alerts as SMARTS (Pfizer LINT filters (42), Glaxo Wellcome Hard Filters (43), Bristol-Myers Squibb HTS Deck Filters (44), NIH MLSMR Excluded Functionality Filters (https://mlsmr.evotec.com/MLSMR_HomePage/pdf/MLSMR_Excluded_Functionality_Filters_200605121510.pdf), University of Dundee NTD Screening Library Filters (45) and Pan Assay Interference Compounds (PAINS) Filters (46), filters derived by Inpharmatica Ltd, set of alerts currently used in SureChEMBL https://www.surechembl.org/knowledgebase/169485). All filters have been run against all ChEMBL compounds for which structures are available and the results included in the database in the COMPOUND_STRUCTURAL_ALERTS, STRUCTURAL_ALERTS and STRUCTURAL_ALERT_SETS tables. These filters can be used to identify compounds that may be problematic in a drug-discovery setting and so guide the selection of compounds and/or help in the interpretation of results. The filters typically represent substructures corresponding to chemically reactive functional groups, those that are associated with toxicity, interfere with certain assay formats or bind promiscuously to targets. Alerts are implemented as reported in the original source but should be interpreted with care, depending on the use-case, and not treated as a blanket filter (e.g. ∼50% of approved drugs have one or more alerts from these sets). These alerts can be viewed on the Compound Report Card via the ChEMBL interface. The alerts are organised by the alert set that a specific functionality is found in and the functional group is highlighted on a depiction of the molecule.

## DATA ACCESS

### The ChEMBL interface

The ChEMBL database is accessible via a basic user interface at: https://www.ebi.ac.uk/chembl. This interface provides core search functionality for compounds (including substructure and similarity searching) and targets as well as keyword searching across assay, cell line and tissue information. Users can also retrieve and filter bioactivity information and browse drug and clinical candidate information (including targets and indications). More details of the user interface and its functionality can be found in previous publications (1,2).

### Downloads and web-services

While the ChEMBL interface provides the functionality necessary for many simple use-cases, some users may prefer to download the database and query it locally (e.g. for use in data mining applications or to integrate with internal data). Each release of ChEMBL is available from our ftp site in a variety of formats including: Oracle, MySQL, PostgreSQL, SQLite, RDF, an SD file of compound structures and a FASTA file of the target sequences, under a Creative Commons Attribution-ShareAlike 3.0 Unported license (http://creativecommons.org/licenses/by-sa/3.0).

The myChEMBL software container is also available (47,48). It is distributed as a virtual machine disk image in many formats such as VMDK, QCOW2 and IMG that can be downloaded using FTP or Vagrant. Alternatively, myChEMBL can be used as a Docker container. The container is based on Ubuntu OS but there exists a CentOS version as well. The container consists of the ChEMBL PostgreSQL database, web services, open source cheminformatics tools (e.g. RDKit: http://www.rdkit.org, OSRA (49) and Open Babel (50)) and Jupyter Notebook tutorials (https://github.com/chembl/mychembl/tree/master/ipython_notebooks), providing a convenient environment for users to interrogate the data.

For users requiring programmatic access to ChEMBL, a comprehensive set of RESTful web services are provided (51), allowing retrieval of ChEMBL data in XML, JSON and YAML formats (see https://www.ebi.ac.uk/chembl/ws for more details and example queries). A Solr-based search functionality is also now available, permitting retrieval of compound, target and assay information via text queries. For example, the following query would retrieve all assays containing the term ‘angiogenesis’: https://www.ebi.ac.uk/chembl/api/data/assay/search?q=angiogenesis. An example Python web service client library is available: https://github.com/chembl/chembl_webresource_client, with usage examples covered in: https://github.com/chembl/mychembl/blob/master/ipython_notebooks/09_myChEMBL_web_services.ipynb.

Web services and myChEMBL are both open source projects and are available from the ChEMBL GitHub repository (https://github.com/chembl/), along with other useful resources to help integration of ChEMBL into software projects (https://arxiv.org/pdf/1607.00378.pdf). They are licensed under an Apache 2 license. Both projects converge to build a new version of the main ChEMBL web interface which will be built on top of web services and deployed and distributed using the myChEMBL container.

### Inclusion of ChEMBL data in other resources

It is often advantageous to integrate ChEMBL data with data from other resources—either to maximise the amount of data that can be retrieved for a particular target/compound, or to provide relevant pharmacology and drug-target data in the context of other data types (e.g. pathway, expression or disease information). ChEMBL data is incorporated into a wide range of other resources including PubChem BioAssay (52), BindingDB (11), CanSAR (53), Open PHACTS (54), Open Targets (55) and the Target Central Resource Database/PHAROS (http://juniper.health.unm.edu/tcrd/, 56), so can also be accessed via these routes. However, since these other resources are different in scope, they do not all incorporate ChEMBL in full (e.g. BindingDB focuses only on binding measurements, while Open Targets incorporates data on drug–target and drug–indication linkage).

## SUMMARY

ChEMBL continues to evolve and grow in order to address an ever-increasing range of use-cases, user communities and applications. The ‘core’ of the resource continues to be molecule–target interaction data curated from the published literature, but as outlined above and in previous reviews (1,2,51,57,58) this has been expanded both in terms of data content, annotation and infrastructure. Many of these developments have been in response to feedback and discussion from ChEMBL's growing user community; we welcome such engagement on the latest version of the resource.
